# An Overview of Target Membrane Proteins for Near-Infrared Photoimmunotherapy

**DOI:** 10.3390/ph18091419

**Published:** 2025-09-21

**Authors:** Motofumi Suzuki, Hirofumi Hanaoka

**Affiliations:** Division of Fundamental Technology Development, Near InfraRed Photo-ImmunoTherapy Research Institute at Kansai Medical University, 2-5-1, Shin-Machi, Hirakata, Osaka 573-1010, Japan; suzukimo@hirakata.kmu.ac.jp

**Keywords:** antibody, cancer treatment, membrane protein, near-infrared photoimmunotherapy

## Abstract

Near-infrared photoimmunotherapy (NIR-PIT) is a recently developed cancer treatment that utilizes antibody–photoabsorber (IRDye700DX [IR700]) conjugates and NIR light. Necrotic cell death associated with lethal membrane damage is induced when this conjugate binds to an antigen on cancer cells, and it is exposed to NIR light. Therefore, various membrane proteins are potential therapeutic targets for NIR-PIT, and many studies have described various target molecules and specific antibodies. To develop future drugs for NIR-PIT, the selection of appropriate target membrane proteins and monoclonal antibodies will be important. In this review, we summarize the membrane targets and antibodies for NIR-PIT used in previous studies, focusing on the characteristics of each molecule.

## 1. Introduction

**Figure 1:**
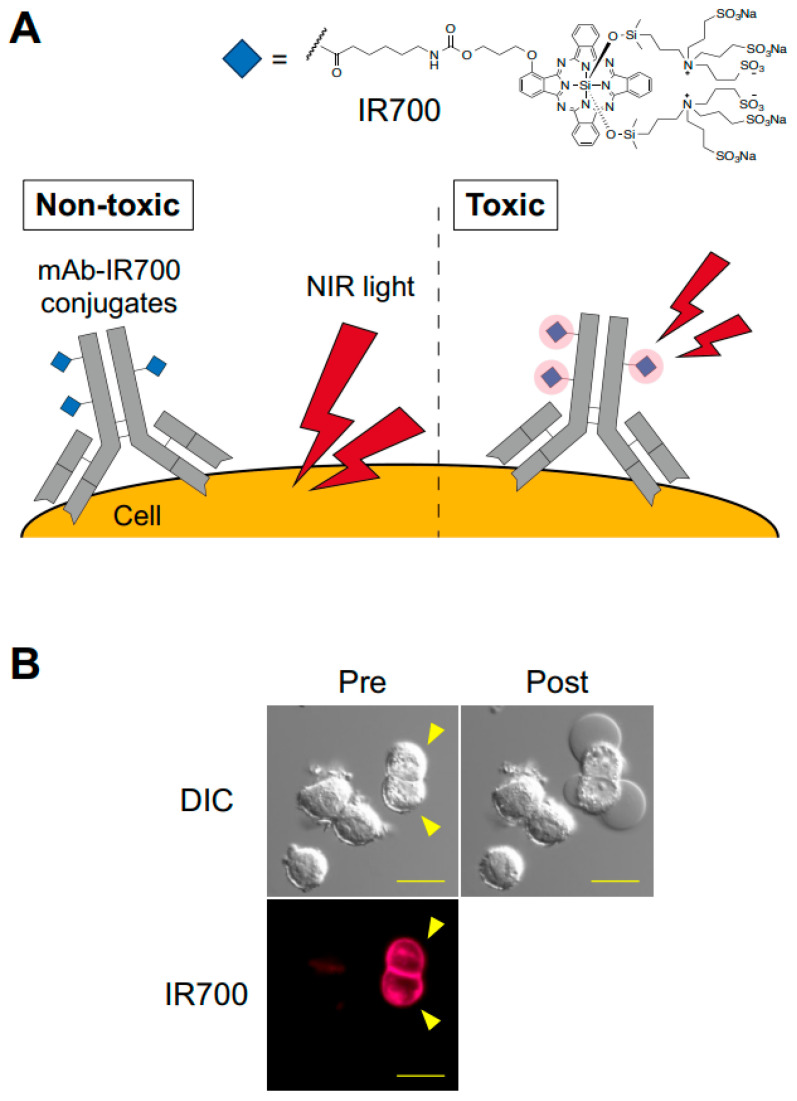
Overview of NIR-PIT. (**A**) NIR-PIT utilizes a photoabsorber (IR700)-conjugated mAb and NIR light. Monotherapy with NIR-PIT agents and NIR light is minimally cytotoxic. Cytotoxicity is induced specifically in agent-bound cells upon light irradiation. (**B**) Representative images of the highly selective cytotoxicity of NIR-PIT. Both targeting protein-expressing and non-expressing cells were co-cultured and treated with NIR-PIT agents. After NIR irradiation, cytotoxic effects were only induced in agent-bound cells (arrowhead).

Near-infrared photoimmunotherapy (NIR-PIT) is a recently developed cancer treatment. The cell-killing effects of NIR-PIT are induced by the photochemical reaction between NIR-PIT agents and NIR light. NIR-PIT agents consist of conjugates of a tumor-targeting monoclonal antibody (mAb) and a photoabsorber (IRDye700DX [IR700]), which absorbs light at 690 nm in the NIR range [1,2]. Although these conjugates and NIR light alone are minimally toxic, lethal damage is induced when this conjugate binds to an antigen on cancer cells and is exposed to NIR light (Figure 1). Thus, NIR-PIT is a highly selective cancer treatment with few adverse effects. Furthermore, NIR-PIT induces immunogenic cell death (ICD) and activates local tumor immunity, meaning that NIR-PIT has the potential to control both the primary tumor and metastatic and recurrent tumors [3,4]. Therefore, NIR-PIT has attracted attention as a novel anti-cancer strategy.

NIR-PIT, using the anti-epidermal growth factor receptor (EGFR) antibody cetuximab conjugated with IR700 (cetuximab sarotalocan sodium, Akalux^®^: Rakuten Medical Inc., San Diego, CA, USA), was approved for clinical use in patients with unresectable locally advanced or recurrent head and neck squamous cell cancer (HNSCC) in Japan [5,6]. In addition, phase III clinical trials in patients with HNSCC are ongoing worldwide [7] (Table 1). To further develop NIR-PIT, it is essential to expand the indications for EGFR-targeting NIR-PIT and develop NIR-PIT using novel targets and antibodies.

Many studies have been performed since the introduction of NIR-PIT, and several review articles on NIR-PIT have been reported [1,8,9]. The mechanism by which NIR-PIT induced cell death is also being elucidated. The structural change in IR700 induced by NIR light exposure causes mAb-IR700 conjugates to aggregate. When this aggregation occurs when mAb is bound to membrane proteins, the cell membrane is damaged, leading to the influx of water into the cell and resulting in cell swelling and eventual cell death. This cell death mode is necrosis-like and differs from apoptosis, which is characterized by cell shrinkage. Thus, the type of antibody-membrane protein complexes formed on the membrane might have a significant impact on the therapeutic efficacy of NIR-PIT, and the properties of the antibody and membrane protein would be greatly involved in the formation of this complex. In addition, because NIR-PIT has a novel therapeutic mechanism, it is necessary to select target molecules and antibodies from a different perspective. This review specifically focuses on the target membrane proteins and the antibodies used in previous studies, which will help guide the future development of drugs for NIR-PIT.

## 2. Membrane Proteins as Targets of NIR-PIT

The cytotoxic mechanism of NIR-PIT is based on cellular membrane damage caused by the photochemical reaction of NIR-PIT agents induced by NIR light and aggregation of the drug–membrane protein complex [10]. Therefore, it is important to summarize the properties of membrane proteins that could influence therapeutic efficacy, such as the type, size, and antibody-binding site of the membrane proteins reported in previous NIR-PIT studies. The involvement of characteristics of membrane proteins in NIR-PIT is discussed in detail in the subsequent sections. In previous reports, the properties of membrane proteins, such as the type, size, and antibody-binding site of the membrane proteins, other than internalization, exert a negligible impact on the therapeutic efficacy of NIR-PIT.

### 2.1. Protein Type

**Figure 2:**
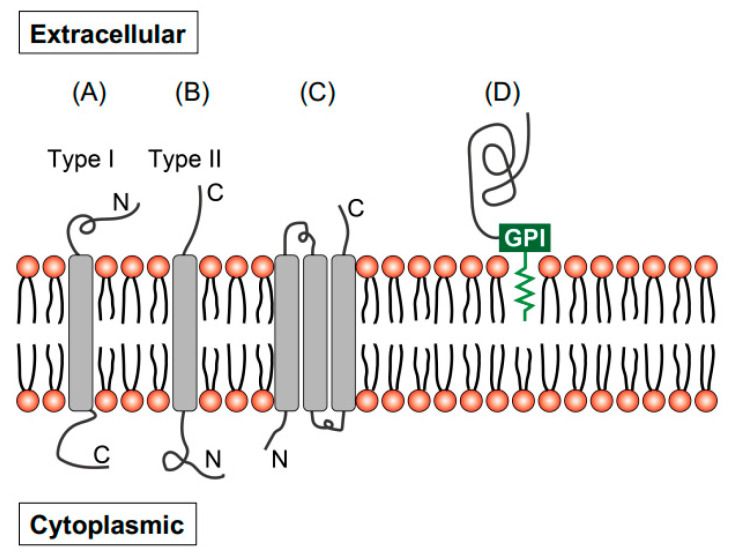
Schematics of membrane proteins. (**A**) Type I transmembrane, (**B**) type II transmembrane, (**C**) multipass transmembrane, and (**D**) GPI-anchored membrane protein.

A large variety of membrane proteins exist in eukaryotic cells, and they can be classified into two groups depending on their membrane interaction: integral and peripheral membrane proteins. In integral membrane proteins, one or multiple domains are embedded within the entire phospholipid bilayer. Conversely, peripheral membrane proteins interact with the membrane through indirect interactions (electrostatic, hydrophobic, and fatty acid modification) or direct interactions (hydrophobic tails or glycosylphosphatidylinositol [GPI] anchors) (Figure 2) [11,12]. Target molecules that have been implemented to date for NIR-PIT are summarized in Table 1. The therapeutic effects of NIR-PIT targeting both integral and peripheral membrane proteins have been reported, although GPI-anchored proteins are not expected to significantly interact with the plasma membrane. Thus, the efficacy of NIR-PIT might be less dependent on the protein type. Of course, several other membrane proteins exist, but it is not necessary to exclude them by type.

### 2.2. Protein Size

The size of membrane proteins, particularly the size of the extracellular domain of the protein, might influence the effect of NIR-PIT because its mechanism of action involves aggregation of the drug–membrane protein complex. The membrane proteins listed in Table 2 vary in size, with the smallest (e.g., podoplanin [PDPN], CD20) being in the 30 kDa range and the largest reaching nearly 200 kDa. In other words, some membrane proteins are slightly larger than IgG antibodies (150 kDa), whereas others are considerably smaller. Although there might be differences in efficacy depending on the size of the membrane protein, the therapeutic effects of NIR-PIT have been observed for small, medium, and large membrane proteins.

Most single-pass transmembrane proteins and GPI-anchored proteins possess a large extracellular domain consisting of several hundred amino acid residues. Among them, PDPN has a relatively small extracellular domain of 130 amino acids [41,42]. The cytotoxic effects of NIR-PIT targeting PDPN with NZ-1 antibody have been reported in malignant pleural mesothelioma [27].

In the case of multipass transmembrane proteins, the size of the extracellular domain is variable. In particular, CD29 and CD133 have large extracellular domains, and they are assumed to respond in the same manner as single-pass transmembrane proteins. Conversely, the extracellular domain of CD20 is shorter than 50 amino acids. However, NIR-PIT using rituximab, an anti-CD20 antibody, exhibited a sufficient therapeutic effect [30]. It is unknown whether NIR-PIT can exert therapeutic effects if the extracellular domain is even smaller, but it is worthy of investigation. However, if the extracellular domain is too small, it might be difficult to develop antibodies that bind to it.

### 2.3. Antibody-Binding Site

Considering membrane damage caused by aggregation of the drug–membrane protein complex, the location at which the antibody binds to the membrane protein is important. If aggregation occurs too distantly from the membrane, then insufficient membrane damage might be induced. Some of the antibodies used in NIR-PIT, listed in Table 1, have undisclosed antigen-binding sites. Considering only cases that are clear, some antibodies bind to sites close to the cell membrane, whereas others bind to more distant sites.

Anti-EGFR antibodies bind slightly away from the membrane, but they have achieved sufficient therapeutic efficacy. In particular, IR700-cetuximab is already in clinical use. Regarding HER2, two anti-HER2 antibodies, namely trastuzumab and pertuzumab, have been evaluated in HER2-targeting NIR-PIT. Trastuzumab binds to the extracellular domain IV of HER2, which is close to the membrane [43]. In contrast, the binding region of pertuzumab is domain II, which is about 300 amino acids away from the membrane [44]. However, the therapeutic effects of NIR-PIT using these antibodies are similar [33]. Further study is needed to clarify the relationship between binding sites and treatment efficacy.

### 2.4. Other Properties

Other properties of membrane proteins might be involved in the therapeutic effect of NIR-PIT. One factor affecting therapeutic efficacy is the internalization of drugs, as the cell-killing effect of NIR-PIT occurs on the membrane surface. It is assumed that the therapeutic effect is better if greater amounts of the drug remain on the membrane surface. Indeed, a previous report suggested that the cytotoxic effects of NIR-PIT with trastuzumab were reduced by internalization of the drug [45]. Conversely, in vivo therapeutic effects have been reported for NIR-PIT using trastuzumab and cetuximab, which are known to be internalized. This might be because the internalization of these antibodies is not rapid, and newly migrated drugs can bind to newly emerging target molecules on the surface of the membrane in vivo; therefore, a certain amount of drugs remains on the cell surface. Thus, membrane proteins that internalize quickly after antibody binding or are slowly recycled might not be suitable targets for NIR-PIT.

The expression levels of membrane proteins on the cell surface are important. If the density of expression is higher, then aggregation among multiple molecules might be induced, leading to greater membrane damage. This is because π-π stacking by phthalocyanine, which occurs in the chemical change of IR700 after light irradiation, is considered important for inducing aggregation, and stacking can occur even between molecules if they are close to each other [46]. Thus, targeting membrane proteins that form dimers or multimers might improve the therapeutic efficacy of NIR-PIT.

Glycosidation or other properties do not appear to significantly affect the therapeutic effect of NIR-PIT. Specific examples are presented in Section 4.

## 3. Antibodies

**Figure 3:**
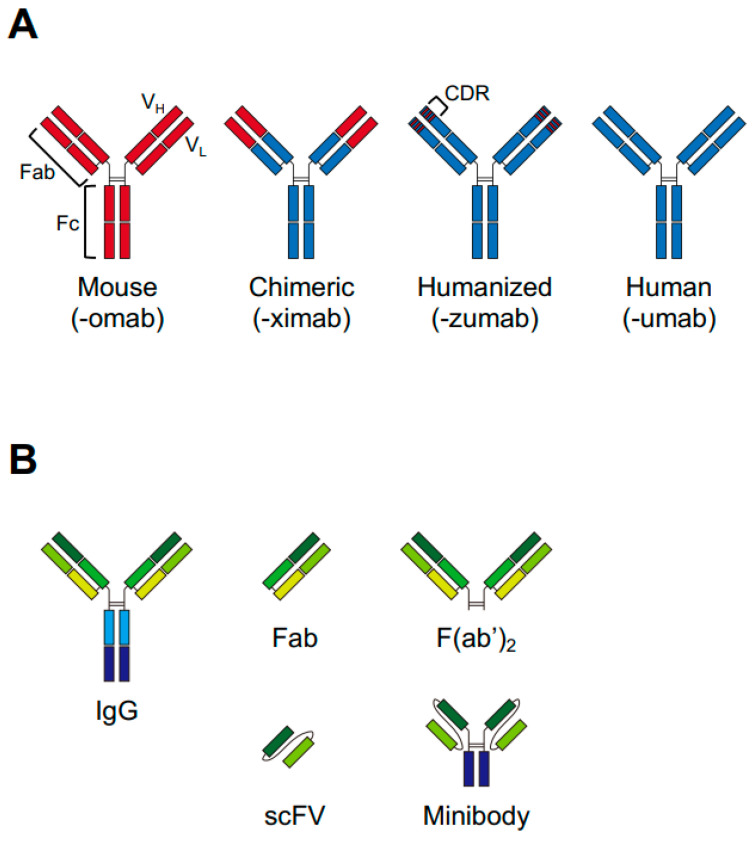
Schematics of the antibody type. (**A**) The structures of different types of monoclonal antibodies. (**B**) Antibody fragment types. CDR, complementarity-determining region; scFV, single-chain antibody variable fragment.

The high selectivity of NIR-PIT is ensured by antibodies. Antibodies are classified into five classes: IgA, IgD, IgE, IgG, and IgM. IgG is the most common isotype in the human serum antibody fraction, accounting for 75% of the circulating antibody pool. IgG is also mainly used as a clinical drug. IgG is further classified by its heavy chain into four subclasses: IgG1, IgG2, IgG3, and IgG4. Each subclass has a different profile, including differences in their binding affinity for crystallizable fragment gamma receptor and half-lives, and the majority of antibodies for tumor treatment are of the IgG1 isotype (Table 3) [47]. Moreover, therapeutic mAbs can be classified into four types by the manufacturing method: murine, chimeric (structural chimeras made by fusing mouse variable regions with human antibody constant regions), humanized (only complementarity-determining region from mice), and fully human antibodies (Figure 3) [48]. Humanization of mAbs can reduce their immunogenicity while maintaining their therapeutic efficacy. The type and subclass of these antibodies affect the therapeutic effects of antibody monotherapy and conjugated mAb therapy, including ADCs, radioimmunotherapy, and immunotoxins.

In NIR-PIT, the properties of the antibody itself have little or no effect on the cytotoxic mechanism. This is because in NIR-PIT, antibodies are used as carriers to deliver IR700 in a cancer-specific manner, and the effect of the antibody itself, such as antibody-dependent cellular cytotoxicity (ADCC) or complement-dependent cytotoxicity (CDC), is not a requirement. Indeed, NIR-PIT with cetuximab (IgG1) and panitumumab (IgG2) induces similar cytotoxic effects in vitro, proving that the IgG subclass has little effect on the cytotoxic mechanism [53]. Of course, the antibody itself is assumed to exert a therapeutic effect in vivo, but even without such an effect, a sufficient therapeutic effect is expected. Therapeutic efficacy has been observed even with IgG4, which induces little ADCC or CDC as previously described. For some target molecules, it might be better to select an antibody without pharmacological effects to avoid side effects in normal organs. Conversely, the biological half-life of antibodies affects their therapeutic efficacy. In mouse models, NIR-PIT employing the fully human antibody panitumumab produces better therapeutic efficacy than that employing the chimeric antibody cetuximab because of the longer biological half-life of panitumumab despite little difference in their in vitro efficacy. In addition, one of the factors affecting the therapeutic efficacy is mAb-IR700 conjugate internalization because the cell-killing effect of photoimmunotherapy is attributable to the aggregation of the drug on the membrane. This opposite is true for ADCs, for which antibody internalization is critical. A previous report suggested that the cytotoxic effects of NIR-PIT are greatest when photochemical reactions occur on the membrane and are attenuated by antibody internalization [45]. Even for the same target molecule, the biological half-life and the internalization rate should be considered when selecting an antibody, which could help optimize NIR-PIT in the future.

The therapeutic effects of NIR-PIT do not require Fc region-mediated effects; therefore, engineered antibody fragments such as those in Figure 3B might be useful as NIR-PIT moieties because of their advantages, such as rapid pharmacokinetics and high tissue penetration compared with whole IgG. Rapid clearance of the drug from the body would reduce concerns about photosensitivity associated with NIR-PIT. High tissue penetration is expected to increase the effectiveness of NIR-PIT by allowing it to reach deeper into the tumor. NIR-PIT using single-chain antibody variable fragments, minibodies, and Fab fragments achieved therapeutic efficacy in vitro and in vivo [21,54,55]. In addition, removal of the Fc region prevents cellular injury induced by ADCC or CDC, thereby avoiding adverse effects outside the target lesion [56]. Thus, fragment antibodies have potential utility in NIR-PIT. However, their short half-lives might affect their therapeutic efficacy as previously mentioned, and further validation is needed, including appropriate IR700 conjugate administration.

## 4. Typical NIR-PIT for Cancer Cells

Typical membrane proteins targeted in NIR-PIT for cancer are described in the subsequent sections. These specific examples illustrate the implementation of NIR-PIT against various targets.

### 4.1. EGFR

EGFR is a 170 kDa transmembrane tyrosine kinase belonging to the erythroblastosis oncogene B (ErbB) family. The EGFR gene encodes a 1186-amino acid transmembrane glycoprotein that consists of three regions: an extracellular ligand-binding domain, a transmembrane region, and an intracellular cytoplasmic tyrosine kinase region [57]. The extracellular domain consists of 621 amino acids, and it is divided into four subdomains, namely domains I (also known as L1, amino acids 1–133), II (also known as S1 or CR1, amino acids 134–312), III (also known as L2, amino acids 313–445), and IV (also known as S2 or CR2, amino acids 446–621) [58,59]. Domains I and III share 37% sequence identity, and they are leucine-rich fragments that interact with ligands [60]. Domain II is a cystine-rich region that contributes to the dimerization of two EGFR moieties [61]. Domain IV is also cystine-rich, and it links to transmembrane domains.

Currently, four EGFR-targeting mAbs, cetuximab (chimeric IgG1 antibody), panitumumab (humanized IgG2 antibody), nimotuzumab (humanized IgG1 antibody), and necitumumab (humanized IgG1 antibody), are already on the market, and they are currently under clinical investigation in various tumors [62]. All of these antibodies inhibit ligand binding to EGFR by binding to domain III, albeit through different binding sites (cetuximab, amino acids 408–468; panitumumab, amino acids 386–391; nimotuzumab, amino acids 353–358; and necitumumab, amino acids 384–409) [62,63]. These differences in binding sites might lead to differences in efficacy and resistance.

In NIR-PIT, the therapeutic efficacy of cetuximab-IR700 (Cet-IR700) and panitumumab-IR700 (Pan-IR700) conjugates was verified by previous studies [9]. The therapeutic efficacy of the Cet-IR700 and Pan-IR700 conjugates has been compared. The in vivo half-life of Pan-IR700 is longer than that of Cet-IR700; thus, NIR-PIT with Pan-IR700 is associated with greater therapeutic efficacy than that with Cet-IR700 [20]. Antibodies other than cetuximab and panitumumab could also be useful in NIR-PIT. A previous report suggested that necitumumab interacts with a similar epitope to cetuximab, although the paratopes are different [64]. Moreover, the paratope cavity of necitumumab is significantly larger than that of panitumumab; thus, necitumumab can bind even cetuximab- and panitumumab-resistant EGFR variants [65,66].

### 4.2. Human Epidermal Growth Factor Receptor 2 (HER2)

HER2, also known as ErbB2, c-erbB2, or HER2/neu, is a 185 kDa transmembrane glycoprotein and a member of the ErbB family. The HER2 gene encodes a 1255-amino acid transmembrane glycoprotein with three regions: an extracellular domain, a transmembrane lipophilic segment, and an intracellular tyrosine kinase domain. The extracellular domain of HER2 consists of 620 amino acids, and it is divided into four domains: two leucine-rich domains (I and III) and two cysteine-rich domains (II and IV). In the ErbB family, the extracellular domain can exist as a tethered form (inactive form) or an extended form (active form). In the tethered form, domain II is tethered to domain IV, rendering it unable to dimerize with other receptors [67,68].

HER2 has been studied as an effective therapeutic target, and two mAbs, namely trastuzumab and pertuzumab, have been developed as HER2-targeting therapies. Trastuzumab is a humanized recombinant monoclonal IgG1 antibody that binds to extracellular domain IV of HER2 [43]. By contrast, the binding region of pertuzumab is domain II, and this drug can inhibit the heterodimerization of HER2 with other receptors [44].

The therapeutic effect of NIR-PIT using trastuzumab-IR700 (Tra-IR700) conjugates has been reported in several tumors, including breast, esophageal, lung, ovarian, and bile duct cancers [2,22,23,69,70,71]. Furthermore, the therapeutic efficacy of NIR-PIT using the combination of Tra-IR700 and pertuzumab-IR700 (Per-IR700) is stronger than that using Tra-IR700 or Per-IR700 alone [24]. In their study, no difference in therapeutic efficacy was observed for NIR-PIT between Tra-IR700 and Per-IR700 in vitro; thus, the region to which the antibody binds might not affect the therapeutic effect.

### 4.3. CD133

CD133, also known as prominin-1 or AC133, is a 97 kDa pentaspan transmembrane glycoprotein that contains two extracellular loops with nine putative N-linked glycosylation sites [72]. After heavy glycosylation, the apparent molecular weight of CD133 increases to 120 kDa. In epithelial cells, CD133 is restricted to plasma membrane protrusions, and it accumulates in membrane microdomains (lipid rafts) [73,74].

The CD133 gene encodes an 865-amino acid transmembrane glycoprotein consisting of an N-terminal extracellular domain (amino acids 20–108), five transmembrane domains, cysteine-rich two small intracellular loops, two large extracellular loops (amino acids 179–433 and 508–792, respectively), and an intercellular cytoplasmic tail [75,76]. The extracellular loop contains nine glycosylation sites (Asn206, Asn220, Asn274, Asn395, Asn414, Asn548, Asn580, Asn729, and Asn730) [72].

To date, the mAbs CD133/1 (AC133) and CD133/2 (AC141), which bind to different glycosylated epitopes in the extracellular domain region of CD133, are the most studied. AC133 conjugated to the genetically modified cytolethal distending toxin AC141, conjugated to polymeric nanoparticles loaded with paclitaxel, and radiolabeled AC133 with iodine-131 exhibit cytotoxic effects in tumors [77,78,79].

NIR-PIT using AC133-IR700 conjugates induces cytotoxicity in glioblastoma stem cells and suppresses tumor growth in both subcutaneous and orthotopic tumor models [33].

### 4.4. CD44

CD44 is a single-chain transmembrane glycoprotein and a member of the cartilage link protein family [80]. In humans, the CD44 gene consists of 19 exons, and the first and last five exons are constant. The intermediate nine exons are subjected to alternative splicing, resulting in multiple CD44 variant (CD44v) isoforms [81]. Accordingly, the molecular weight of CD44 ranges from 85 to 230 kDa. The smallest CD44 isoform (85–95 kDa), which is only coded by the constant exons, is most common, and it is called standard CD44 (CD44s). CD44s is ubiquitously expressed on the membranes of vertebrate cells, and it plays essential roles in homeostasis [82]. By contrast, CD44v expression is mostly restricted to epithelia [83]. Importantly, CD44v isoforms are cancer stem cell markers, and they are associated with tumor progression and metastasis [84].

All CD44 isoforms consist of one extracellular domain, one transmembrane domain, and one intracellular domain [85]. Several ligands, including hyaluronic acid (HA) and osteopontin, bind to the extracellular domain and regulate the PI3K signaling pathway. CD44s is a 363-amino acid glycoprotein that consists of a 270-amino acid extracellular domain, a 21-amino acid transmembrane domain, and a 72-amino acid C-terminal cytoplasmic domain. The theoretical molecular weight of 37 kDa is increased to 85–95 kDa through the attachment of glycosaminoglycan.

CD44 is an attractive target, and more than 140 commercially available CD44-targeting mAbs have been developed [86]. H4C4 is an anti-human CD44 IgG1 mAb that reduces tumor growth, metastasis, and post-radiation recurrence in pancreatic cancer [87]. IM7, another anti-mouse/human pan-CD44 IgG2b mAb that recognizes the HA-binding domain of CD44, inhibits the cell migration and invasion of breast cancer cells [88,89]. Information on other antibodies is omitted from this review.

NIR-PIT using IM7 conjugated with IR700 has been reported. NIR-PIT targeting CD44 induces ICD and increases tumor-infiltrating CD8^+^ cell counts in syngeneic models of colorectal, lung, head and neck, and oral cancers [90,91,92,93,94].

### 4.5. Carcinoembryonic Antigen (CEA)

CEA, also known as CEA-related cell adhesion molecule 5 (CEACAM5) or CD66e, is a 150–200 kDa heavily glycosylated protein. CEA belongs to the immunoglobulin (Ig) superfamily, and it is expressed on the cellular membrane through a GPI anchor [95]. CEA belongs to the CEACAM family, which is a subgroup of the CEA family. It was first isolated from human colorectal cancer tissue, and it is commonly used in clinical practice as a serum biomarker [96].

CEA consists of 651 amino acids and contains seven Ig-like domains, including a single Ig variable region-like N-terminal domain followed by six Ig constant region type 2-like domains termed A1, B1, A2, B2, A3, and B3 [97,98,99]. The C-terminal domain of CEA is anchored to the cellular membrane through GPI [100].

In normal colon epithelial cells, CEA expression is strictly localized to the apical surface. Conversely, CEA is expressed over the entire cell surface of colorectal cancer cells [101]. Moreover, CEA is overexpressed in various tumors, including breast, pancreatic, lung, and thyroid tumors [102,103,104,105]. CEA is a GPI-anchored protein that has no intracellular domain. While the anti-CEA mAbs are useful as imaging carriers [106,107], but antibodies alone are unlikely to show therapeutic efficacy [108]. Anti-CEA mAbs have potential as antibody-drug conjugates (ADCs), and labetuzumab govitecan (IMMU 130), an anti-CEA/SN-38 conjugate, displayed therapeutic activity in heavily pretreated patients with metastatic colorectal cancer [109]. Moreover, several cytotoxic agents based on anti-CEA mAbs, such as PR1A3 and 15-1-32, have been developed [108,110,111].

NIR-PIT targeting CEA using chimeric anti-CEA mAb (Genara Biosciences LLC, Morgan Hill, CA, USA) or the anti-CEA IgG4 C2-45 suppressed tumor progression in gastric and pancreatic tumor models [35,112,113]. Moreover, NIR-PIT targeting CEA was effective in a patient-derived orthotopic xenograft model [34].

### 4.6. Prostate-Specific Membrane Antigen (PSMA)

PSMA, also known as glutamate carboxypeptidase II or N-acetylated alpha-linked acidic dipeptidase in the central/peripheral nervous system, is a type II transmembrane glycoprotein that is highly expressed in prostate cancer cells [114]. The predicted molecular weight of PSMA is 84 kDa, but it is increased to more than 100 kDa by glycosylation, which is necessary for proteolytic activity [115,116]. In addition, PSMA is expressed on the cell surface in both dimeric and monomeric forms, and dimerization is required for its enzymatic activity [117].

PSMA consists of a short N-terminal cytoplasmic domain (19 amino acids), a single hydrophobic transmembrane domain (24 amino acids), and a large extracellular domain (707 amino acids) [118]. The extracellular domain has nine N-glycosylation sites, two domains of unknown function (amino acids 44–150 and 151–274, respectively), proline- and glycine-rich regions (amino acids 145–172 and 249–273, respectively), a catalytic domain (amino acids 274–587), and a helical dimerization domain (amino acids 587–750) [115,119].

7E11, also known as CYT-356 and capromab, is the first developed anti-PSMA mAb, and [^111^In]-capromab pendetide has been used as a single-photon emission computed tomography agent in patients with prostate cancer [120,121]. However, clinical trials using [^90^Y]-capromab pendetide for radioimmunotherapy were discontinued because of poor efficacy [122]. Molecular mapping revealed that the reason for the limited therapeutic efficacy is that the 7E11 recognizes the intracellular domain of PSMA [123,124]. To improve the imaging quality and therapeutic efficacy of the PSMA-targeting strategy, several mAbs targeting the extracellular epitope of PSMA have been developed, and J591 (humanized IgG1 mAb) is the most commonly used anti-PSMA antibody for radioimmunotherapy in clinical trials [125].

NIR-PIT targeting PSMA employing a commercially available antibody (clone: YPSMA-1) induces cytotoxic effects associated with membrane damage [126]. Moreover, NIR-PIT using a fully human anti-PSMA mAb that binds a conformation-dependent epitope within the extracellular domain significantly suppresses tumor proliferation [29].

### 4.7. Podoplanin (PDPN)

PDPN, also known as T1α, aggrus, or gp36, is a 38 kDa type I transmembrane mucin-like glycoprotein. PDPN is expressed in normal tissue, such as kidney podocytes, lung type I alveolar epithelium, and lymphatic endothelium [127,128,129]. PDPN has no enzymatic activity motif, and it performs its function through interactions with other proteins. PDPN is an endogenous ligand for C-type lectin-like receptor 2, which is highly expressed in platelets.

PDPN consists of an N-terminal extracellular domain of 130 amino acids, followed by a single transmembrane domain of 25 amino acids, and its intracellular domain consists of only nine amino acids [41,42]. The extracellular domain contains a repeat sequence of EDxxVTPG, known as PLAG1, 2, 3, and PLAG-like domain (PLD or PLAG4) [130,131].

Currently, several anti-PDPN mAbs have been developed, including NZ-1, P2-0, and MS-1 (recognizing the PLAG2/3 domain); D2-40 (recognizing the PLAG1/2 domain); LpMab-2 (recognizing the glycopeptide Thr55–Leu64 of human PDPN); LpMab23 (recognizing the naked peptide Gly54–Leu64 of human PDPN); and chPMab and 2F7 (recognizing PLD) [131]. Some of them can inhibit PDPN-induced lung metastasis [132,133,134,135]. Moreover, chimeric antigen receptor-transduced T cells targeting PDPN using NZ-1 have displayed efficacy against glioblastoma in vitro and in orthotopic models [136].

The cytotoxic effects of NIR-PIT targeting PDPN with NZ-1 have been reported in malignant pleural mesothelioma [27]. Moreover, NIR-PIT induced death in PDPN-expressing cancer cells and cancer-associated fibroblasts, leading to an increase in the number of cytotoxic T cells in tumor tissue [137].

### 4.8. Intercellular Adhesion Molecule-1 (ICAM-1)

ICAM1, also known as CD54, is a 90 kDa Ig superfamily transmembrane protein. The molecular weight of human ICAM-1 varies up to 114 kDa because it contains eight putative N-glycosylation sites [138].

ICAM-1 consists of five extracellular Ig-like domains (D1–D5), a short hydrophobic transmembrane region, and a small carboxyl-terminal cytoplasmic tail that interacts with the actin cytoskeleton [139].

The therapeutic effects of NIR-PIT targeting ICAM-1 in triple-negative breast cancer have been reported [25]. As an NIR-PIT agent, a commercially available anti-ICAM-1 mAb (clone R6-5-D6) has been employed. Interestingly, drastic histological changes, namely cytoplasmic vacuolation and proliferation marker downregulation, are induced only 2 h after in vivo NIR-PIT targeting ICAM-1.

### 4.9. Nectin Cell Adhesion Molecule 4 (Nectin-4)

Nectin-4, also known as poliovirus receptor-related protein 4, is a 66 kDa Ca^2+^-independent Ig-like type I transmembrane protein. Nectin-4 is a member of Nectin family, which regulates cellular movement, proliferation, survival, differentiation, and polarization [140,141]. Although Nectin 1–3 are widely expressed in adult tissues, the expression of Nectin-4 is limited to the embryo and placenta [142,143].

Nectin-4 consists of an extracellular region, a transmembrane region, and a cytoplasmic tail that interacts with afadin, an F-actin-associated molecule, to bind to the actin cytoskeleton [144]. The extracellular domain contains two Ig-like C2-type domains and one Ig-like V-type domain.

The fully anti-human Nectin-4 mAb AGS-22M6, targeting the extracellular domain of human Nectin-4, was generated using the XenoMouse strains genetically engineered with a humanized humoral immune system [145]. Enfortumab vedotin, which consists of AGS-22M6 conjugated with monomethyl auristatin E, has been developed, and its therapeutic efficacy has been reported in various tumor models, including breast, bladder, pancreatic, and lung cancers [146]. The US Food and Drug Administration has approved enfortumab vedotin-ejfv (Padcev, Astellas Pharma) in combination with pembrolizumab (anti-PD1 mAb) for the treatment of patients with locally advanced or metastatic urothelial cancer who are ineligible for cisplatin-containing chemotherapy [147].

The cytotoxic effects of NIR-PIT targeting Nectin-4 using enfortumab biosimilars in luminal subtype bladder cancer cell lines have been reported [26]. Moreover, NIR-PIT has displayed efficacy in bladder cancer orthotopic models. Previously, the effectiveness of endoscopic NIR-PIT was reported; therefore, NIR light irradiation using cystoscopy might be effective and clinically available.

## 5. Limitation

This review concentrates on representative membrane proteins as well as those membrane proteins that have not been addressed in previous reviews, with an emphasis on the classifications of membrane proteins and the associated antibodies that may be utilized in NIR-PIT agents. Consequently, it does not encompass all proteins and antibodies that are relevant to NIR-PIT. Conversely, it encapsulates the categorization of membrane proteins and antibodies, which will prove advantageous for forthcoming investigations into NIR-PIT. Second, there are no reports of cases where NIR-PIT treatment was ineffective, so it is unclear which membrane proteins are not applicable to. However, NIR-PIT currently works on various types of membrane proteins and may be effective against all types of membrane proteins. Further knowledge will be accumulated as it is applied to various membrane proteins in the future.

## 6. Summary

In the decade since NIR-PIT was developed, its efficacy against various cancer types using different antibodies has been reported. Currently, only NIR-PIT using the EGFR antibody cetuximab is approved for clinical use. To apply NIR-PIT in additional cancer regimens, it is important to identify an appropriate set of cancer types and targeting proteins and develop useful antibodies. The cytotoxic mechanism of NIR-PIT is based on cellular membrane damage caused by the photochemical reaction of agents induced by NIR light and aggregation of the drug–membrane protein complex. Therefore, to attain sufficient efficacy for NIR-PIT, it appears important to select appropriate membrane proteins and antibodies. However, as previously mentioned, the therapeutic effects of NIR-PIT on various types of membrane proteins using all types of antibodies have been demonstrated. The properties of membrane proteins, such as the type, size, and antibody-binding site of the membrane proteins, exert a negligible impact on the therapeutic efficacy of NIR-PIT. Therefore, a variety of target molecules can be selected as long as they are sufficiently present on the cell membrane. It may be more suitable when expressed at high density. On the other hand, membrane proteins that internalize quickly after antibody binding and are slowly recycled might not be suitable targets. That is, if a target cell overexpresses a molecule on the cell surface and produces antibodies against it, then the cell can potentially be killed by NIR-PIT. NIR-PIT does not cause cytotoxicity without light exposure, so if the antibody itself has no pharmacological effect, it might be possible to target more molecules than ever before, since it is not limited to those expressed only in cancer. Indeed, specificity could be achieved by application of light to the cancer tissue only. In addition, because the pharmacological effect of the antibody itself is not required and accumulation at sites distant from the target lesion is not a problem, the development of antibodies could be easier than that for other antibody-based drugs. From the viewpoint of side effects, the pharmacological effects of the antibody itself must be considered. NIR-PIT has potential as a novel anti-cancer strategy because it is a highly effective and less invasive tumor treatment, and thus, further research and development are expected.

## Figures and Tables

**Table 1:** Clinical trials of NIR-PIT.

Clinical Trial	Patients	NIR-PIT	Results	Ref.
Phase I/IIa	Male: 24, Female: 6	1−4 cycles	CR: 4 (13.3%), PR: 9 (30%), SD: 11 (36.7%), DC: 24 (80%), PD: 6 (20%)	[7]
Phase I	Female: 3	1 cycles	CR: 0 (0%), PR: 2 (66.7%), SD: 0 (0%), DC: 2 (66.7%), PD: 1 (33.3%)	[5]

**Table 2:** Target molecules for photoimmunotherapy targeting cancer cells [5,13,14,15,16,17,18,19,20,21,22,23,24,25,26,27,28,29,30,31,32,33,34,35,36,37,38,39,40].

Protein Type	Target Molecule	IR700 Carrier	Cancer	Refs.
Type 1 single-pass transmembrane protein	Cadherin-17	ARB102	Pancreatic cancer	[13]
	CD44	IM7	Breast cancer, colon cancer, oral cancer, lung cancer	[14]
	CD146	YY146	Melanoma	[15]
	c-KIT	12A8	Gastrointestinal stromal tumors	[16]
	DLL3	Rovalpituzumab	Lung cancer	[17]
	EGFR	Cetuximab, panitumumab	Head and neck cancer, bladder cancer, breast cancer, lung cancer, liver cancer	[5,18,19,20]
	GPA33	A33ScFv	Colon cancer	[21]
	HER2	Trastuzumab, pertuzumab	Breast cancer, gastric cancer, ovarian cancer	[22,23,24]
	ICAM1	R65-D6	Breast cancer	[25]
	Nectin-4	Enfortumab biosimilar	Bladder cancer	[26]
	Podoplanin	NZ-1	Mesothelioma	[27]
	TROP2	HuT6-16-2	Pancreatic cancer	[28]
Type 2 single-pass transmembrane protein	PSMA	YPSMA-1	Prostate cancer	[29]
Multi-pass transmemnrane protein	CD20	Rituximab	B cell lymphoma	[30]
	CD29	KMI6	Melanoma	[31]
	CD47	B6H12	Bladder cancer	[32]
	CD133	AC133	Glioblastoma	[33]
GPI-anchored protein	CEA	Chimeric anti-CEA mAb, M5A	Pancreatic cancer, colon cancer	[34,35,36]
	Glypican-1	Miltuximab	Prostate cancer, bladder cancer, brain cancer, ovary cancer	[37]
	Glypican-3	YP7, HN3	A431/G1 cells (epidermoid cancer cells stably expressing human GPC3)	[38,39]
	Mesothelin	hYP218	A431/H9 cells (epidermoid cancer cells stably expressing human mesothelin)	[40]

**Table 3:** IgG isotype for photoimmunotherapy agents, PMID: [49,50,51,52].

Target	Antibody	Type	Isotype	Ref.
CEA	Labetuzumab	Humanized	IgG1	[49]
CD44v6	Bivatuzumab	Humanized	IgG1	[50]
EGFR	Cetuximab	Chimeric	IgG1	[51]
	Necitumumab	Fully human	IgG1	[51]
	Nimotuzumab	Humanized	IgG1	[51]
	Panitumumab	Fully human	IgG2	[51]
HER2	Pertuzumab	Humanized	IgG1	[51]
	Trastuzumab	Humanized	IgG1	[51]
ICAM-1	Bersanlimab	Fully human	IgG1	[52]
Nectin-4	Enfortumab	Fully human	IgG1	[51]

## Data Availability

No new data were created or analyzed in this study.

## Abbreviations

CDR	complementarity-determining region
CEA	carcinoembryonic antigen
EGFR	epidermal growth factor receptor
ErbB	erythroblastosis oncogene B
GPI	glycosylphosphatidylinositol
HER2	human epidermal growth factor receptor
HNSCC	head and neck squamous cell cancer
ICAM-1	intercellular adhesion molecule-1
ICD	immunogenic cell death; mAb, monoclonal antibody
Nectin-4	Nectin cell adhesion molecule 4
NIR-PIT	near-infrared photoimmunotherapy
PDPN	podoplanin
PSMA	prostate-specific membrane antigen
scFV	single-chain antibody variable fragment
